# Deblur Rapidly Resolves Single-Nucleotide Community Sequence Patterns

**DOI:** 10.1128/mSystems.00191-16

**Published:** 2017-03-07

**Authors:** Amnon Amir, Daniel McDonald, Jose A. Navas-Molina, Evguenia Kopylova, James T. Morton, Zhenjiang Zech Xu, Eric P. Kightley, Luke R. Thompson, Embriette R. Hyde, Antonio Gonzalez, Rob Knight

**Affiliations:** a Department of Pediatrics, University of California San Diego, La Jolla, California, USA; b Department of Applied Mathematics, and Interdisciplinary Quantitative Biology Graduate Program, University of Colorado Boulder, Boulder, Colorado, USA; c Department of Computer Science and Engineering, University of California San Diego, La Jolla, California, USA; d Center for Microbiome Innovation, University of California San Diego, San Diego, California, USA; Argonne National Laboratory

**Keywords:** DNA sequencing, microbiome

## Abstract

High-throughput sequencing of 16S ribosomal RNA gene amplicons has facilitated understanding of complex microbial communities, but the inherent noise in PCR and DNA sequencing limits differentiation of closely related bacteria. Although many scientific questions can be addressed with broad taxonomic profiles, clinical, food safety, and some ecological applications require higher specificity. Here we introduce a novel sub-operational-taxonomic-unit (sOTU) approach, Deblur, that uses error profiles to obtain putative error-free sequences from Illumina MiSeq and HiSeq sequencing platforms. Deblur substantially reduces computational demands relative to similar sOTU methods and does so with similar or better sensitivity and specificity. Using simulations, mock mixtures, and real data sets, we detected closely related bacterial sequences with single nucleotide differences while removing false positives and maintaining stability in detection, suggesting that Deblur is limited only by read length and diversity within the amplicon sequences. Because Deblur operates on a per-sample level, it scales to modern data sets and meta-analyses. To highlight Deblur’s ability to integrate data sets, we include an interactive exploration of its application to multiple distinct sequencing rounds of the American Gut Project. Deblur is open source under the Berkeley Software Distribution (BSD) license, easily installable, and downloadable from https://github.com/biocore/deblur.

**IMPORTANCE** Deblur provides a rapid and sensitive means to assess ecological patterns driven by differentiation of closely related taxa. This algorithm provides a solution to the problem of identifying real ecological differences between taxa whose amplicons differ by a single base pair, is applicable in an automated fashion to large-scale sequencing data sets, and can integrate sequencing runs collected over time.

## Abstract (precis)

Deblur provides a rapid and sensitive means to assess ecological patterns driven by differentiation of closely related taxa. This algorithm provides a solution to the problem of identifying real ecological differences between taxa whose amplicons differ by a single base pair, is applicable in an automated fashion to large-scale sequencing data sets, and can integrate sequencing runs collected over time.

## OBSERVATION

An important goal of microbiome research is identifying taxa present in a given sample. Next-generation sequencing of the 16S rRNA gene on Illumina instruments is commonly used for this task but suffers from an error rate of 0.1% per nucleotide (1). In a typical study spanning millions of sequences, many sequences contain at least one error, obscuring the underlying biology through inaccurate taxon identification and inflated diversity statistics. These errors seldom affect statistical tests for differences between two communities, but higher precision is becoming increasingly important as the field moves toward applications with clinical or regulatory significance.

**FIG 1:**
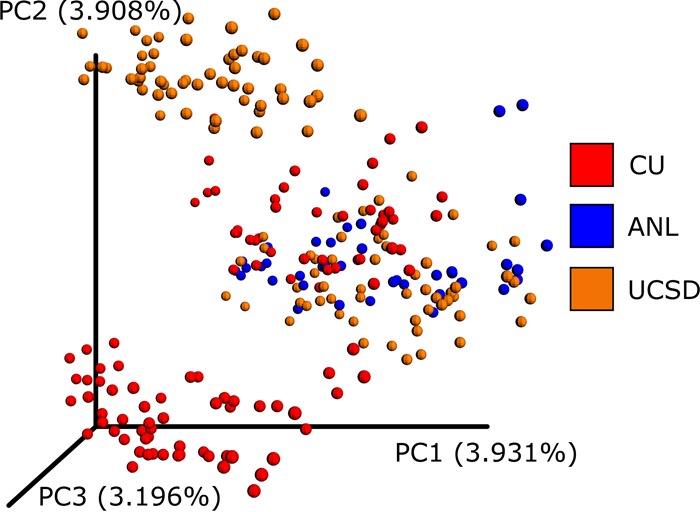
A principal-coordinate analysis plot of UniFrac distances from *de novo* OTUs as visualized by Emperor. A subset of American Gut Project samples spanning sequencing centers and rounds were selected. UCLUST (3) was run independently per round via QIIME. The resulting OTU tables were merged, normalizing sequencing identifiers (IDs) such that if the same sequence was observed in multiple rounds it would receive the same ID. Observations with fewer than 10 counts were dropped. The data were rarefied to 5,000 sequences per sample. The plot shown is based on unweighted UniFrac distances, and the samples are colored by the sequencing center. An interactive visualization can be viewed at https://nbviewer.jupyter.org/github/knightlab-analyses/deblur-manuscript/blob/master/embedded_figure_1.ipynb; the coloring used in the static image can be done by selecting “run_center” as the scatter field. CU, University of Colorado Boulder; ANL, Argonne National Laboratory; UCSD, University of California San Diego.

The classic approach to overcoming these errors is to cluster amplicon sequences into operational taxonomic units (OTUs) (2, 3) based on an arbitrary sequence identity threshold. This approach reduces problems caused by erroneous sequences but also reduces phylogenetic resolution because sequences below the identity threshold cannot be differentiated. Furthermore, OTUs picked within individual data sets cannot be merged when different centroid sequences are chosen in each data set, limiting the ability to combine OTU results. This problem of merging OTUs is exemplified by Fig. 1, for which *de novo* OTUs were assessed independently on separate rounds (i.e., “run_center”) of sequencing with a subset of American Gut Project data. Although approaches such as closed-reference and open-reference OTU picking (4) reduce this problem, integrating large data sets into a single OTU space remains a challenge. Here we describe Deblur, a novel sub-OTU (sOTU) method for fast and accurate identification of exact sequences in amplicon studies, and show how it can be used to integrate large data sets.

Similar in concept to AmpliconNoise (5), a denoising method for pyrosequencing, Deblur, like DADA2 (6) and UNOISE2 (7), attempts to obtain single-nucleotide resolution from Illumina data with statistical methods to infer the putative true sequences within a sample that give rise to the distribution of observed error-prone sequences. Unlike DADA2 and UNOISE2, Deblur operates on each sample independently. It compares sequence-to-sequence Hamming distances within a sample to an upper-bound error profile (see Table S1 in the supplemental material; mathematical derivation in Text S1 in the supplemental material) combined with a greedy algorithm to obtain single-nucleotide resolution. The Deblur algorithm is implemented as follows (see Fig. S1 in the supplemental material). First, sequences are sorted by abundance. Second, from the most to least abundant sequence, the number of predicted error-derived reads is subtracted from neighboring reads based on their Hamming distance, using an upper bound on the error probability. A parameterized maximal probability for indels (defaulting to 0.01) and a parameterized mean read error rate for normalization (defaulting to 0.5%) are included. Finally, any sequence whose abundance drops to 0 after a subtraction is removed from the list of valid sequences. Sequences not considered to be valid (i.e., noise) are removed. After applying Deblur, only reads likely to have been presented to the sequencer are retained. However, it is possible that the reads would still contain chimeras originating from PCR. Reads are filtered for *de novo* chimeras using UCHIME (8) as implemented by VSEARCH (9) using modified parameters (Text S1).

10.1128/mSystems.00191-16.1 TEXT S1  Details on materials and methods and experimental design. Download TEXT S1, DOCX file, 0.1 MB. Copyright © 2017 Amir et al. 2017 Amir et al. This content is distributed under the terms of the Creative Commons Attribution 4.0 International license.

10.1128/mSystems.00191-16.2 FIG S1  The Deblur pipeline. A demultiplexed and quality filtered fasta/fastq file (or a directory of per-sample FASTA/FASTQ files) is used as the input to the pipeline. Following initial splitting to per-sample fasta files, all processing is done independently on each sample. Sequences are trimmed and dereplicated with singletons removed. Reads are then depleted from sequencing artifacts either using a set of known sequencing artifacts (such as PhiX) (negative filtering) or using a set of known 16S sequences (positive filtering). Resulting nonartifact reads are then aligned for easy indel detection. This multiple sequence alignment is then used as the input for the Deblur algorithm. Each Deblurred sample is then checked for *de novo* chimeras, and the resulting sOTUs from all samples are combined into a single BIOM (20) table (with sequences labeled as the sOTU IDs). Download FIG S1, PDF file, 0.8 MB. Copyright © 2017 Amir et al. 2017 Amir et al. This content is distributed under the terms of the Creative Commons Attribution 4.0 International license.

10.1128/mSystems.00191-16.5 TABLE S1  The full error profile used by Deblur. Download TABLE S1, XLSX file, 0.03 MB. Copyright © 2017 Amir et al. 2017 Amir et al. This content is distributed under the terms of the Creative Commons Attribution 4.0 International license.

Using simulated, mock, and real data sets, we compared the performance of Deblur with the performance of DADA2 and UNOISE2. Most analyses using real data sets did not complete using the free version of UNOISE2. We omitted classic OTU methods and MED (10), given the benchmarks described in reference 6. Finally, we applied Deblur to multiple sequencing rounds of the American Gut Project samples to demonstrate integration across sequencing runs from multiple instruments.

**FIG 2:**
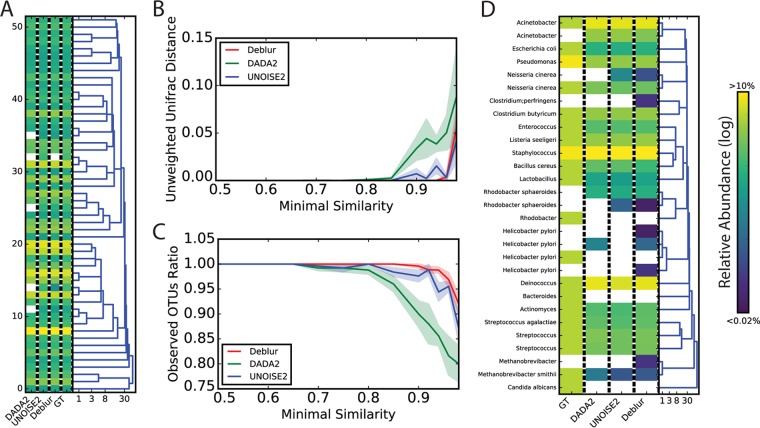
Benchmarks of OTU picking tools on artificial communities. (A) A simulation was performed on the basis of samples from a real fecal community (11) using the 52 most abundant bacterial species identified in this study. Reads were then simulated using an ART Illumina (12) read simulator. OTU picking was performed on these simulated reads using UNOISE2, DADA2, and Deblur. The relative abundances predicted by each of these tools and the ground truth (GT) are shown in the heat map. The dendrogram was built using hierarchical clustering based on the Hamming distance between the sequences, with numbers indicating sequence similarity (log scale). (B) Simulated communities with various levels of sequence-sequence similarity. Unweighted UniFrac distances of the predicted OTUs from UNOISE2, DADA2, and Deblur were compared to those of the original composition of the simulated communities. The *x* axis denotes the similarity radius for each community. The shaded area denotes the standard error of the mean distance estimation (based on 10 random repeats per community). (C) Similar to panel B but with the ratio of observed OTUs (predicted by UNOISE2, DADA2, and Deblur) to actual OTUs in each simulation indicated. (D) Performance of Deblur, UNOISE2, and DADA2 on the even1 community from mock-3 (14). GT data denote the expected ground truth relative frequency for each sOTU as informed by the design of the mock community. Dendrograms and colors are the same as described for panel A.

We first compared methods using simulated communities based on bacterial taxa and frequencies obtained from Sanger sequencing of one stool sample (11). Reads were simulated from this real community using ART (12) to produce Illumina-like sequence data. All three methods identified sOTUs with single-nucleotide differences (Fig. 2A). We then simulated sequences over increasing levels of similarity between the real sequences, measuring unweighted UniFrac (13) distances to ground truth, and observed OTUs (Fig. 2B and C). Deblur, DADA2, and UNOISE2 were all close to the ground truth except at high similarity levels, but all three suffered from limitations in distinguishing the true reads. Using the MiSeq data generated from a 22-member community (“mock-3”) (14) trimmed to the first 150 nucleotides (nt) due to poor 3′ quality, we observed that all methods produced results that were close to the ground truth (Fig. 2D).

**FIG 3:**
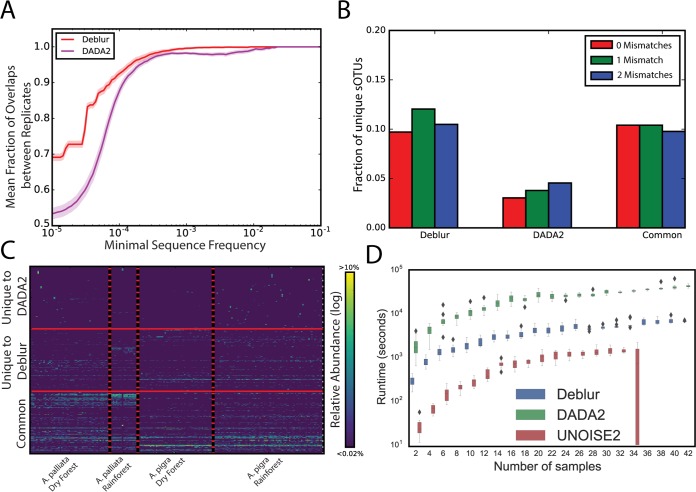
Benchmarks of OTU picking tools on natural communities. (A) Stability analysis on experimental technical repeats. Data indicate fractions of overlapping sOTUs from two technical replicates in all OTUs as a function of the minimal frequency threshold present in one of the repeats. (B and C) Application of Deblur in the howler monkey data set. (B) Fraction of sequences matching entries in the NCBI nr/nt database (as of 1 December 2016) with 0.1 or 2 mismatches (red, green, or blue, respectively) from sOTUs unique to Deblur or to DADA2 or present in both (left to right). (C) Heat maps showing sOTUs (rows) in common with Deblur and DADA2, as well as those unique to Deblur and DADA2 (bottom, middle, and top rows, respectively). Samples (columns) are sorted by species and habitat. A total of 200 sOTUs per group (i.e., common, unique to Deblur, or unique to DADA2) were randomly selected for visualization purposes. (D) Single-threaded runtime comparison of Deblur, DADA2, and UNOISE2 against one of the stability MiSeq runs at increasing numbers of samples.

**FIG 4:**
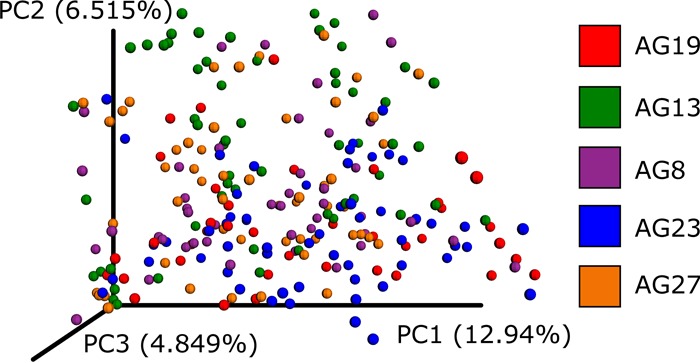
A principal-coordinate analysis plot of UniFrac distances from Deblur as visualized by Emperor. A subset of American Gut Project samples spanning sequencing centers and rounds were selected. Each sample was processed separately by Deblur. Observations with fewer than 10 counts were dropped. The data were rarefied to 5,000 sequences per sample. The plot shown is based on unweighted UniFrac distances and is colored according to the round of sequencing in the American Gut Project (AG). An interactive visualization can be viewed at https://nbviewer.jupyter.org/github/knightlab-analyses/deblur-manuscript/blob/master/embedded_figure_4.ipynb; the coloring used in the static image can be made by selecting the “center_project_name” as the scatter field.

**FIG 5:**
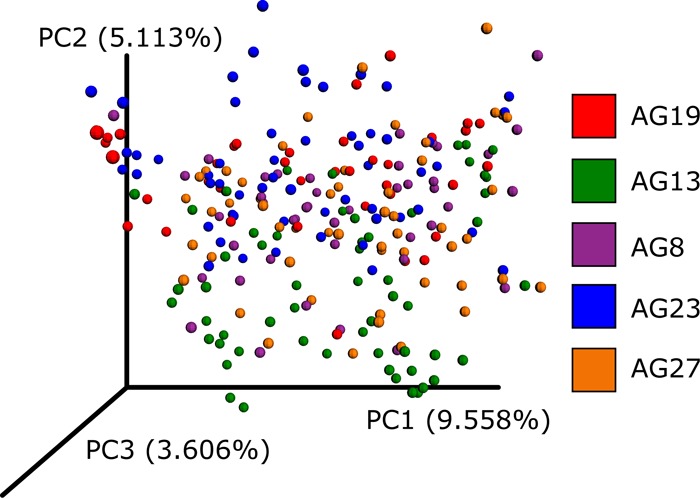
A principal-coordinate analysis plot of UniFrac distances from UNOISE2 as visualized by Emperor. A subset of American Gut Project samples spanning sequencing centers and rounds were selected. UNOISE2 was run independently per round. The resulting sOTU tables were merged, normalizing sequencing IDs such that if the same sequence were observed in multiple rounds it would receive the same ID. Observations with fewer than 10 counts were dropped. The data were rarefied to 5,000 sequences per sample. The plot shown is based on unweighted UniFrac distances and is colored according to the round of sequencing in the American Gut Project. An interactive visualization can be viewed at https://nbviewer.jupyter.org/github/knightlab-analyses/deblur-manuscript/blob/master/embedded_figure_5.ipynb; the coloring used in the static image can be made by selecting the “center_project_name” as the scatter field. The static shot is oriented to show PC1 versus PC2, and the separation is more pronounced if orienting the projection to look at PC2 versus PC3.

Stability (i.e., obtaining the same sOTU across different samples) is becoming critical as more study designs exploit existing samples from resources like the Earth Microbiome Project (15) or require integration of sequence data collected over time such as the American Gut Project (http://americangut.org). We compared the levels of stability of Deblur and DADA2 using technical replicates from a data set consisting of 40 individuals, each with one fecal sample sequenced twice on two separate MiSeq runs (16). sOTUs for each run were assessed separately, and we compared the fractions of sOTUs from one run to those present in the second run, as a function of the minimal sOTU frequency. Deblur showed greater stability than DADA2 at a higher frequency cutoff (Fig. 3A), indicating that a larger fraction of sOTUs from the first run were also identified in the second run. To further test the stability of Deblur, we sourced previously sequenced fecal samples from the American Gut Project and selected fecal samples which spanned five distinct sequencing runs over multiple instruments from three geographically distinct locations (Table S2). As can be seen in the Emperor (17) plot of a principal-coordinate analysis of unweighted UniFrac distances, samples from different sequencing rounds (denoted by “center_project_name”) are integrated in the ordination, demonstrating that the sequencing rounds do not separate as is typical with OTU-based methods (Fig. 4). In contrast, performing the same experiment with UNOISE2 (running it per round and merging results with respect to commonly identified sequences) produced an observable effect mediated by the sequencing round and run center (Fig. 5), although the magnitude of the effect was notably less than that observed with *de novo* OTUs (Fig. 1). We note that this is in contradiction to the recommended way of running UNOISE2 (i.e., we did not run it on the full data set); however, we are unaware of a mode of operation (free or full version) capable of operating on modern large-scale data sets.

10.1128/mSystems.00191-16.6 TABLE S2  Samples from the American Gut Project used for an integration, highlighting the sequencing round, sequencing location, and date of sequencing. Download TABLE S2, XLSX file, 0.1 MB. Copyright © 2017 Amir et al. 2017 Amir et al. This content is distributed under the terms of the Creative Commons Attribution 4.0 International license.

Next, we compared DADA2 and Deblur using a complex natural community and a previously published data set of fecal samples from two species of howler monkeys (18). Deblur and DADA2 detected 1,938 and 1,636 sOTUs, respectively, after removal of sOTUs with fewer than 10 total reads from each method (Fig. S2A). Following filtering, about 70% of the sOTUs were identical between the methods (Fig. S2B). As expected, both methods identified differential sOTUs (permutation-based rank mean test; 0.1 false-discovery rate–Benjamini-Hochberg method [FDR-BH] control value) with 61% of Deblur sOTUs differentiating between primate species (1,193/1,938), compared to 55% of DADA2 sOTUs (891/1,636). To assess whether the sOTUs unique to either method were from increased numbers of artifacts, we used BLAST (19) to compare each unique sequence against nt/nr and plotted the fraction of sOTUs with zero, one, or two mismatches. We observed that sOTUs unique to Deblur showed fewer mismatches than those unique to DADA2 (Fig. 3B). The distribution of sOTUs over the monkey samples suggests that the sOTUs unique to Deblur are more plausible because they show a pattern similar to those identified by both methods, whereas the sOTUs unique to DADA2 have markedly different patterns of clusters of unique sOTUs within single samples (Fig. 3C).

10.1128/mSystems.00191-16.3 FIG S2  Howler monkey sOTUs identified by Deblur and DADA2. Reads from the howler monkey data set (18) are shown. (A and B) sOTUs identified per method (A) and their abundance (B). The fractions of overlapping sOTUs under conditions of increasing filtering based on minimum total read counts per sOTU are indicated. Download FIG S2, PDF file, 0.1 MB. Copyright © 2017 Amir et al. 2017 Amir et al. This content is distributed under the terms of the Creative Commons Attribution 4.0 International license.

Finally, to explore performance characteristics, we used a MiSeq run from the stability analysis in order to assess computational space and time demands of DADA2, Deblur, and UNOISE2 (where possible) over an increasing number of samples. UNOISE2 was an order of magnitude faster than Deblur, while Deblur was an order of magnitude faster than DADA2 (Fig. 3D). Deblur maintained a fairly flat memory profile (Fig. S3) in contrast to the growth observed with DADA2 and UNOISE2.

10.1128/mSystems.00191-16.4 FIG S3  Space characterization. Memory consumption on random subsets of one MiSeq run from reference 16 is indicated. (A) Memory use in kilobytes for Deblur, DADA2, and UNOISE2 over a log scale (B). Detail of memory use of Deblur in megabytes using a linear scale. Download FIG S3, PDF file, 0.9 MB. Copyright © 2017 Amir et al. 2017 Amir et al. This content is distributed under the terms of the Creative Commons Attribution 4.0 International license.

Like DADA2 and UNOISE2, which approach the same concept with different algorithms, Deblur produces stable sOTUs which can achieve single-nucleotide resolution and which can be used in place of OTUs. However, unlike DADA2 and UNOISE2, Deblur does not require operation on the full study and can thus be parallelized easily to very large projects. Finally, Deblur is released under the Berkeley Software Distribution (BSD) open source license, allowing easy commercial adoption and peer scrutiny. Consequently, because of its stability, ability to integrate, performance, and open-source license, Deblur is positioned to operate on present and future large-data sets as well as continued discovery through reuse of existing rich data sets.
