# Supplementary material for: Deblur Rapidly Resolves Single-Nucleotide Community Sequence Patterns
Source: mSystems. 2017 Mar 7;2(2):e00191-16. doi: 10.1128/mSystems.00191-16 (PMC5340863; doi:10.1128/mSystems.00191-16)
Supplement: TEXT S1 [file sys002172091s4.docx]

**Supplemental**

**Materials and methods**

**Experimental design**

**Detailed mathematical formation**

**American Gut Project Integration**

**Supplemental references**

**Materials and methods**

*The Deblur pipeline*

The Deblur pipeline steps are illustrated in fig. S1. Per-sample reads are trimmed to a constant parameter controlled length (shorter reads are discarded) and dereplicated using VSEARCH (1), with singleton reads removed. Reads are then filtered for non-amplicon sequences through one of two user selectable ways: negative filtering by removing all reads matching a FASTA database (by default, composed of PhiX and sequencing adapters) or positive filtering by comparison to a database of known sequences (by default the Greengenes 13_8 88% OTUs (2)). Both filtering options are implemented using SortMeRNA v2.0 (3). By default, Deblur uses a negative filtering mode. Following filtering, the reads are then aligned with MAFFT (4) to construct a multiple sequence alignment (MSA). This MSA is used to detect indels, and is the input to the greedy Deblur algorithm, which is implemented as follows: sequences are first sorted based on their abundance; then, iterating from the most to least abundant sequence, the number of predicted error-derived reads (as assessed by the upper error rate bound and a constant probability of 0.01 for an indel, combined with the mean read error rate for normalization) are subtracted from all neighboring reads (a neighbor is defined as within a Hamming distance of 11); finally, any read whose frequency drops to 0 following subtraction is removed from the list of valid sequences. A more detailed mathematical formulation is provided at the end of this document. Following the application of the Deblur algorithm, only reads which are highly likely of being presented to the sequencer are retained. However, it is possible that the reads still contain chimeras originating from PCR amplification prior to sequencing. Therefore, the reads are additionally filtered for *de novo* chimeras using VSEARCH with non-default parameters: --dn=0.000001, --xn=1000, --minh=10000000, --mindiffs=5. These non-default parameters translate into indicating the presence of a chimera if:

$$\frac{Y_{L}}{{10}^{3}\left( N_{L}+{10}^{-6} \right)+A_{L}}\times\frac{Y_{R}}{{10}^{3}\left( N_{R}+{10}^{-6} \right)+A_{R}}>{10}^{7}$$

Using these parameters leads to not identifying a sequence as a chimera if there is one or more No of Abstain vote (i.e. if both parts of the chimera don’t exactly match other sequences in the deblurred fasta). These parameters are used since we assume post-deblur sequences are the actual sequences in the sample, so all chimeras must originate from these exact sequences. This final output sequence set represents the predicted true sequences and their frequency in a sample. These per-sample results are then aggregated into a BIOM (5) table, where the sequences serve as the observation IDs. For the results presented in the paper, unless indicated otherwise, Deblur was run with all parameters set to the default values with the exception of trim length, which was set to the appropriate run read length, and use of negative filtering mode (-n) for negative mode (for removal of PhiX and adapter sequences).

*Determination of default read error parameters for Deblur*

The error profile for amplicon sequencing depends on both PCR and sequencing errors, and can depend on the local sequence, PCR polymerase and number of cycles, sequencing run and cycle (6). While PHRED scores give an estimate of the sequencing derived error, they cannot account for PCR derived errors. Additionally, the errors are not necessarily independent leading to the probability that 2 errors in the same sequence can be higher than the square of the probability of one error. Therefore, Deblur uses an upper bound on the error profile as a function of the Hamming distance. Using a variety of internal experiments with known spike in, as well as mock mixtures from (7) and two large validation studies currently (manuscripts in preparation), we can get an estimate of the read error distribution and upper bounds. These conservative high bounds were picked to minimize false positive sequences (i.e. reducing the number of erroneous sequences), at the expense of increasing the number of possible false negatives (i.e. real sequences that are removed as errors by Deblur). Because of the Deblur algorithm, we anticipate that false negatives will only occur if there are very close true sequences which vary a lot in their frequency, or if the sequence count of the true sequence is below threshold.

The values chosen as default are: 0.06 for the upper bound on the probability of acquiring a single nucleotide mismatch, decreasing to 0.02 for two mismatches and further decreasing down to 0.0005 at 11 mismatches (see Table S1 for full default error profile). Indels have a constant probability of 0.01. Additionally, since the number of observed reads for a real sequence is reduced by read errors, and the fraction of such reads depends on the read length, a mean read error rate of 0.5% per nucleotide is used for normalizing the fraction of reads prior to subtraction, which is approximated by 1-(1-mean_error)^read_len^.

*Comparison to other methods*

In order to enable direct comparison between Deblur and the other methods tested, similar read length and processing steps were applied as follows: per-sample reads were trimmed to constant length (reads below the trim length were discarded), and for unsimulated data, reads were quality filtered using QIIME 1.9.1 split_libraries_fastq.py with a quality threshold of 19. Specific parameters for each method used are described below:

Deblur v0.1.5:

Deblur was run using the default negative filtering mode (removing only phiX and adapter artifact sequences) All default parameter values were used unless explicitly noted.

DADA2 v1.0.3 (8):

DADA2 was run with defaults using the “run_dada.R” script from (9). This is a wrapper script to facilitate command line interaction with DADA2 and written by the authors of DADA2.

UNOISE2 v9.1.13 (10):

UNOISE2 was run following the recommendations on the homepage (11) with the exception that the “mergepairs” step was not performed. Additionally, all commands were passed “-threads 1” to avoid implicit parallelization.

*Runtime comparisons*

The A739F run from the repeats dataset (12) was used. Data were demultiplexed using QIIME 1.9.1 with using a quality threshold of 19. Data were trimmed to 150nt prior to execution; all reads < 150nt were dropped. This resulted in 42 samples, with a mean of 292,541 sequences per sample (standard deviation of 120,680).

Samples were randomly selected without replacement at each step 10 times. A step width of 2 was used over the range [2, 42]. Each method (Deblur, DADA2, UNOISE2; parameters and versions noted above) was run on the resulting selection of samples. All methods were run with a single thread. Timing and memory information was tracked using GNU Time v1.7

GNU Time v1.7 has a known bug in which maximum memory is reported at 4x the amount. A correction for this was performed after the fact.

All executions were performed using independent jobs on a Torque-based compute cluster composed of Intel E5-2640 v3 2.60GHz processors. Data were staged to local filesystems prior to job execution, and all intermediary files generated were written to local filesystems. Stage in and stage out are not included in runtime characterizations.

**American Gut Project integration**

Sample information was obtained from (13). The following exclusion criteria were applied to the samples. First, any non-fecal sample was dropped. Second, any sample with > 100 reads previously denoted as a bloom by the American Gut Project were omitted. Third, any sample with fewer than 10,000 reads following a classic closed reference OTU picking were dropped. Last, an individual must be part of the SUBSET_HEALTHY. From the remaining set of samples, we selected the top 5 rounds based on the number of remaining samples. The sample IDs were then used to query EBI to retrieve the raw sequence data. The sequence data were trimmed to 150nt, and any read under 150nt was dropped.

For embedded figure 1, the samples were run through UCLUST in *de novo* mode via QIIME 1.9.1 per-round using default parameters. The resulting per-round OTU tables were merged such that, if an identical sequence was found in multiple rounds, it received a common identifier. OTUs with fewer than 10 reads were dropped.

For embedded figures 4, the samples were then Deblurred using default parameters as noted above with the exception that sOTUs with fewer than 10 reads were dropped.

For embedded figure 5, the samples were run through UNOISE2 per-round using the parameters as noted above. The resulting per-round OTU tables were merged such that, if an identical sequence was found in multiple rounds, it received a common identifier. sOTUs with fewer than 10 reads were dropped.

The resulting tables were rarefied to 5,000 sequences per sample. The remaining sOTUs were aligned with defaults using PyNAST (14) via QIIME 1.9.1’s (15) align_seqs.py, the alignment was filtered with defaults using filter_alignment.py and a *de novo* phylogeny constructed from the resulting alignment using FastTree 2.1.3 (16). Unweighted UniFrac (17) and subsequent principal coordinates were then computed using QIIME’s beta_diversity.py and principal_coordinates.py respectively and with defaults. Finally, a visualization was constructed using EMPeror 1.0.0beta5 (18).

**Experimental design**

A variety of previously published datasets and techniques were used to assess the quality of the Deblur method. We did not use the mock data from (19) as the URL provided in the manuscript resulted in a HTTP 404 error (accessed on November 18, 2016). We did not use the “extreme” data from (8) as only processed data were available from SRA and we were unable to obtain the raw reads. Specific details per figure are listed below:

*Figure 1A (Simulation based on a real fecal community)*

Complete 16S sequences (obtained via Sanger sequencing of human fecal samples (20) were retrieved from Genbank (accessions EU761594–EU768801). Sequences were trimmed to the V4 region pulling out 150bp fragments (using a custom script aligning to the 515f primer), and sequences present at least 25 times were used for the simulation (totaling 52 sequences). These sequences and their corresponding frequencies were used for simulating Illumina reads with ART (21) with the default parameters and 50 reads per sequence. The resulting reads were used as input to the various OTU picking methods.

*Figure 1B,C (Simulated communities):*

For each simulation, a seed sequence was randomly selected from the complete Greengenes dataset reduced to the V4 region 150bp reads. An additional 25 random bacteria or archaea were selected under the constraint of having a similarity to the seed sequence within a threshold (x-axis in figure 2B, C). Frequencies for the 26 bacteria were taken from a Power-law distribution 1/sqrt(n). 10,000 reads were simulated using ART with default parameters. OTU tables were then generated from the various methods, and unweighted UniFrac distances (17) were calculated using QIIME 1.9.1 (15) and compared to the ground truth, using *de novo* trees constructed using PyNAST 0.1 (14) and FastTree 2.1.3 (16) via QIIME 1.9.1 (15).

Figure 1D (mock community):

Reads from mock community DS2 were obtained from (22)(7). For creation of the ground truth sequences, sequences of the strains listed in community B were obtained from SILVA (23) using the best matching strain when present. If the exact strain was not found, the sequence of the species was used instead. Raw counts per sequence were obtained by counting the number of reads in the original FASTA file corresponding to the given sequence.

*Figure 2A (Stability analysis)*

Reads from two MiSeq runs of technical repeats from 40 fecal samples and two samples described as noise were obtained from (12). Each sample was rarefied to 100,000 reads, and then each run was processed independently with Deblur and DADA2. For every minimal frequency threshold (x-axis figure 2A), every OTU present below the threshold in the first run was tested to see if it existed in the second run, and the fraction of such OTUs present in both samples is plotted in the y-axis.

*Figure 2B,C (Application of Deblur in the Howler monkey dataset):*

Reads from two Howler monkey species (24) were obtained from Qiita (study 10315). Following sOTU identification using Deblur and DADA2 as described above, OTUs with <10 reads total (in all samples combined, after normalizing to 10000 reads/sample) were removed from further analysis. For identification of differentially abundant OTUs between the two species, we used a rank-mean permutation test followed by a Benjamini-Hochberg FDR control at level 0.1. To assess the number of sOTUs matching closely to plausible bacterial sequences, sOTUs were BLASTed against nr/nt using megablast default parameters, except the following: max target sequences: 10, expect threshold: 0.1, word size 32. For the heat map (C), 200 sOTUs from each group (common to both Deblur and DADA2, unique to Deblur and unique to DADA2) were randomly chosen. The sOTUs within each group were ordered using single linkage clustering (Euclidean distance on mean of 0 and standard deviation of 1 scaled reads per sOTU).

**Detailed mathematical formulation**

*Definitions*

We begin with a set of *reads* {*r_i_*}, lists of length *N*_bases_ whose elements are bases in {*A,C,G,T*}, and a corresponding set of *counts* {*c_i_*}, such that *c*_I_ is the number of instances of the read *r_k_* in the original sample. The experiment can be conceived of as a stochastic process in which each instance of each read in the original sample is sequenced, base by base, and where there is some probability that for each base, the experiment will make a mistake and return the wrong base. If we knew the exact probability for every location, base, read, and mistake, we could then compute the probability that the experiment will return any particular read given any other read. We don’t know these probabilities exactly, and so instead we use approximate quantities in our computations. We define an *error* to be an event in which a base is misread by the experiment as any other base (a similar approach is also applied to insertions/deletions). A *misread* is an event in which at least one error is made by the experiment in sequencing a read. Let *α* be the mean probability of a misread.

Errors are not i.i.d., as they can depend on the specific sequence, on the position within the read, and on the general goodness of the read. Therefore, working only with the average misread probability *α* can lead to a lot of misreads passing as good reads. We are therefore also interested in some reasonable upper bound on the probability of getting a misread with exactly *k* errors.

For a threshold *T* and for a number of mismatches k denote *β_T_*(*K*) the upper bound on the error probability, such that for *T* of the reads in the original samples, the probability of obtaining a misread with exactly *k* errors is lower than β*_T_*(*k*). Note that choosing this threshold *T* involves a type-1/type-2 balance; e.g., setting it to *T* = 100% may lead to a very high value of *β_T_*(*k*), which can prevent identification of real sequences (see below). For Deblur, the upper bound default values are based on statistics collected from several MiSeq and HiSeq runs, and are not calculated separately for each experiment processed. The upper bound is denoted as *β*(*k*).

*Algorithm Description*

The output of the experiment is a set of reads {*r_i_*} and counts {*c_i_*}, with *i* = 1, 2, ..., N_reads_. We assume that the sets are indexed so that the *c_i_* are decreasing. We know that some of the reads that should have been *r_1_* were sequenced as other reads; therefore, we want a correction to *c_1_*. Let *c′_1_* be the actual count of *r_1_* in the original sample; we know that the experiment will make some mistakes sequencing the *c′_1_* instances of *r_1_*, and we want to increase *c_1_* accordingly to account for this. As a rough first pass, we use the mean probability of obtaining a misread, *α,* to approximate the original count *c′_1_* by:

$c^{'}\cong c_{1}\frac{1}{1- \alpha}.$ (1)

Given this rough approximation of *c′_1_*, we will now reduce the counts of other reads because we expect that some of them are in fact misreads of *r_1_*. Let *d_jk_* be the Hamming distance between two reads *r_j_* and *r_k_*. Then we expect (with 95% confidence, or whatever threshold we chose for the *β*(*k*)), no more than *β*(*d_1k_*)·*c′_1_* of the original *c′_1_* counts of *r_1_* to have been misread as *r_k_*, where *r_k_* is any other read. This is a strong upper bound; it is likely that many fewer instances of *c′_1_* were misread as *r_k_*, but because there are more of *r_1_* than there are of *r_k_*, we favor *r_1_* over *r_k_*. We therefore go through each of the *r_k_* and adjust their counts:

$c_{k}\leftarrow\left[ 1- \beta\left( d_{1k} \right) \right]{c'}_{1}.$ (2)

If the right-hand-side is negative, we set *c_k_* to 0 instead. The algorithm then continues with the next read *r_2_* etc., until all reads have been processed.

*Algorithm pseudocode*

def deblur(Reads, Counts, alpha, Betas):

# Assumes Reads and Counts are sorted by Counts.

# iterate through the reads :

NReads = length (Reads , Counts)

For i in NReads :

# increase c because we know there were mistakes

# Note: this increase is NOT used in updating c,

# only in reducing the other counts

cprime i = Counts[i] / ( 1 − alpha)

# iterate through the remaining reads.

# Note: because we only loop over j > i, preceding counts

# will not be reduced by future ones.

for j>i, j<=NReads:

# compute the Hamming distance

d = HammingDistance( Reads[ i ] , Reads[ j ] )

# choose the upper bound on the error based on

# this distance

beta = Betas [d]

# shrink c j by the appropriate amount of cprime i

Counts[j] = Counts[j] − (cprime i ∗ (1 − beta))

# don’t let it be negative

if Counts[j] < 0: Counts[j] = 0

return Counts

Following the Deblur algorithm, we expect the majority (*T*) of the misreads to be removed, thus cleaning the reads from errors.

*Limitations of the Deblur algorithm*

The amount of type-1 sequences (misreads still present after Deblur) depends on the upper bound strictness (*T*) for the given run.

Type-2 errors (true reads present in the sample which were removed) can also occur. Let *r_1_*,*r_2_* be true sequences in the sample, with corresponding number of reads *c_1_* ≥ *c_2_*. We denote d the hamming distance between *r_1_* and *r_2_*, and if f *c_2_ / c_1_* < *β*(*d*), then read *r_2_* may be removed from the resulting sequences (as it lies below *c_1_* the noise limit with regard to read *r_1_*). However, this error is limited to close sequences which have a large difference in their relative frequencies.

Note that for obtaining fast performance, Deblur is implemented as a greedy and not an exact algorithm. The order in which the sequences are sorted can affect the resulting frequencies (since the algorithm only changes sequences down the list). However, this has a very limited effect, since the change in frequency is limited by *β*(*k*)^2^ and *β*(*k*) ≤ 0.1; therefore, it is expected to have a minor effect on the resulting frequencies.

**Supplementary References:**

1. **Rognes T**, **Flouri T**, **Nichols B**, **Quince C**, **Mahé F**. 2016. VSEARCH: a versatile open source tool for metagenomics. PeerJ Prepr **4**:e2409v1.

2. **McDonald D**, **Price MN**, **Goodrich J**, **Nawrocki EP**, **DeSantis TZ**, **Probst A**, **Andersen GL**, **Knight R**, **Hugenholtz P**. 2012. An improved Greengenes taxonomy with explicit ranks for ecological and evolutionary analyses of bacteria and archaea. ISME J **6**:610–618.

3. **Kopylova E**, **Noe L**, **Touzet H**. 2012. SortMeRNA: fast and accurate filtering of ribosomal RNAs in metatranscriptomic data. Bioinformatics **28**:3211–3217.

4. **Katoh K**, **Standley DM**. 2013. MAFFT multiple sequence alignment software version 7: improvements in performance and usability. Mol Biol Evol **30**:772–780.

5. **McDonald D**, **Clemente JC**, **Kuczynski J**, **Rideout JR**, **Stombaugh J**, **Wendel D**, **Wilke A**, **Huse S**, **Hufnagle J**, **Meyer F**, **Knight R**, **Caporaso JG**. 2012. The Biological Observation Matrix (BIOM) format or: how I learned to stop worrying and love the ome-ome. Gigascience **1**:7.

6. **Schirmer M**, **Ijaz UZ**, **D’Amore R**, **Hall N**, **Sloan WT**, **Quince C**. 2015. Insight into biases and sequencing errors for amplicon sequencing with the Illumina MiSeq platform. Nucleic Acids Res **43**.

7. **Bokulich NA**, **Rideout JR**, **Mercurio WG**, **Shiffer A**, **Wolfe B**, **Maurice CF**, **Dutton RJ**, **Turnbaugh PJ**, **Knight R**, **Caporaso JG**. 2016. mockrobiota: a Public Resource for Microbiome Bioinformatics Benchmarking. mSystems **1**:e00062-16.

8. **Callahan BJ**, **McMurdie PJ**, **Rosen MJ**, **Han AW**, **Johnson AJA**, **Holmes SP**. 2016. DADA2: High-resolution sample inference from Illumina amplicon data. Nat Methods **13**:581–3.

9. **Callahan BJ**. run_dada.R script. Retrieved November 18, 2016, from https://github.com/qiime2/q2-dada2/blob/master/q2_dada2/assets/run_dada.R.

10. **Edgar RC**. UNOISE2 : improved error-correction for Illumina 16S and ITS amplicon sequencing.

11. **Edgar RC**. UNOISE2 pipeline. Retrieved November 18, 2016, from http://www.drive5.com/usearch/manual/unoise_pipeline.html.

12. **Hildebrand F**, **Tadeo R**, **Voigt AY**, **Bork P**, **Raes J**. 2014. LotuS: an efficient and user-friendly OTU processing pipeline. Microbiome **2**:30.

13. **Consortium AGP**. American Gut Project data (July 29 2016). Retrieved November 18, 2016, from ftp://ftp.microbio.me/AmericanGut/ag-July-29-2016/.

14. **Caporaso JG**, **Bittinger K**, **Bushman FD**, **DeSantis TZ**, **Andersen GL**, **Knight R**. 2010. PyNAST: a flexible tool for aligning sequences to a template alignment. Bioinformatics **26**:266–267.

15. **Caporaso JG**, **Kuczynski J**, **Stombaugh J**, **Bittinger K**, **Bushman FD**, **Costello EK**, **Fierer N**, **Pena AG**, **Goodrich JK**, **Gordon JI**, **Huttley GA**, **Kelley ST**, **Knights D**, **Koenig JE**, **Ley RE**, **Lozupone CA**, **McDonald D**, **Muegge BD**, **Pirrung M**, **Reeder J**, **Sevinsky JR**, **Turnbaugh PJ**, **Walters WA**, **Widmann J**, **Yatsunenko T**, **Zaneveld J**, **Knight R**. 2010. QIIME allows analysis of high-throughput community sequencing data. Nat Methods **7**:335–336.

16. **Price MN**, **Dehal PS**, **Arkin AP**. 2010. FastTree 2--approximately maximum-likelihood trees for large alignments. PLoS One **5**:e9490.

17. **Hamady M**, **Lozupone C**, **Knight R**. 2010. Fast UniFrac: facilitating high-throughput phylogenetic analyses of microbial communities including analysis of pyrosequencing and PhyloChip data. ISME J **4**:17–27.

18. **Vazquez-Baeza Y**, **Pirrung M**, **Gonzalez A**, **Knight R**. 2013. EMPeror: a tool for visualizing high-throughput microbial community data. Gigascience **2**:16.

19. **Kozich JJ**, **Westcott SL**, **Baxter NT**, **Highlander SK**, **Schloss PD**. 2013. Development of a dual-index sequencing strategy and curation pipeline for analyzing amplicon sequence data on the miseq illumina sequencing platform. Appl Environ Microbiol **79**:5112–5120.

20. **Dethlefsen L**, **Huse S**, **Sogin ML**, **Relman DA**. 2008. The pervasive effects of an antibiotic on the human gut microbiota, as revealed by deep 16S rRNA sequencing. PLoS Biol **6**:e280.

21. **Huang W**, **Li L**, **Myers JR**, **Marth GT**. 2012. ART: a next-generation sequencing read simulator. Bioinformatics **28**:593–4.

22. **Bokulich N a**, **Subramanian S**, **Faith JJ**, **Gevers D**, **Gordon JI**, **Knight R**, **Mills D a**, **Caporaso JG**. 2013. Quality-filtering vastly improves diversity estimates from Illumina amplicon sequencing. Nat Methods **10**:57–9.

23. **Pruesse E**, **Quast C**, **Knittel K**, **Fuchs BM**, **Ludwig W**, **Peplies J**, **Glockner FO**. 2007. SILVA: a comprehensive online resource for quality checked and aligned ribosomal RNA sequence data compatible with ARB. Nucleic Acids Res **35**:7188–7196.

24. **Amato KR**, **Martinez-Mota R**, **Righini N**, **Raguet-Schofield M**, **Corcione FP**, **Marini E**, **Humphrey G**, **Gogul G**, **Gaffney J**, **Lovelace E**, **Williams L**, **Luong A**, **Dominguez-Bello MG**, **Stumpf RM**, **White B**, **Nelson KE**, **Knight R**, **Leigh SR**. 2016. Phylogenetic and ecological factors impact the gut microbiota of two Neotropical primate species. Oecologia **180**:717–33.
