# Supplementary figures and images for: Deblur Rapidly Resolves Single-Nucleotide Community Sequence Patterns
Source: mSystems. 2017 Mar 7;2(2):e00191-16. doi: 10.1128/mSystems.00191-16 (PMC5340863; doi:10.1128/mSystems.00191-16)

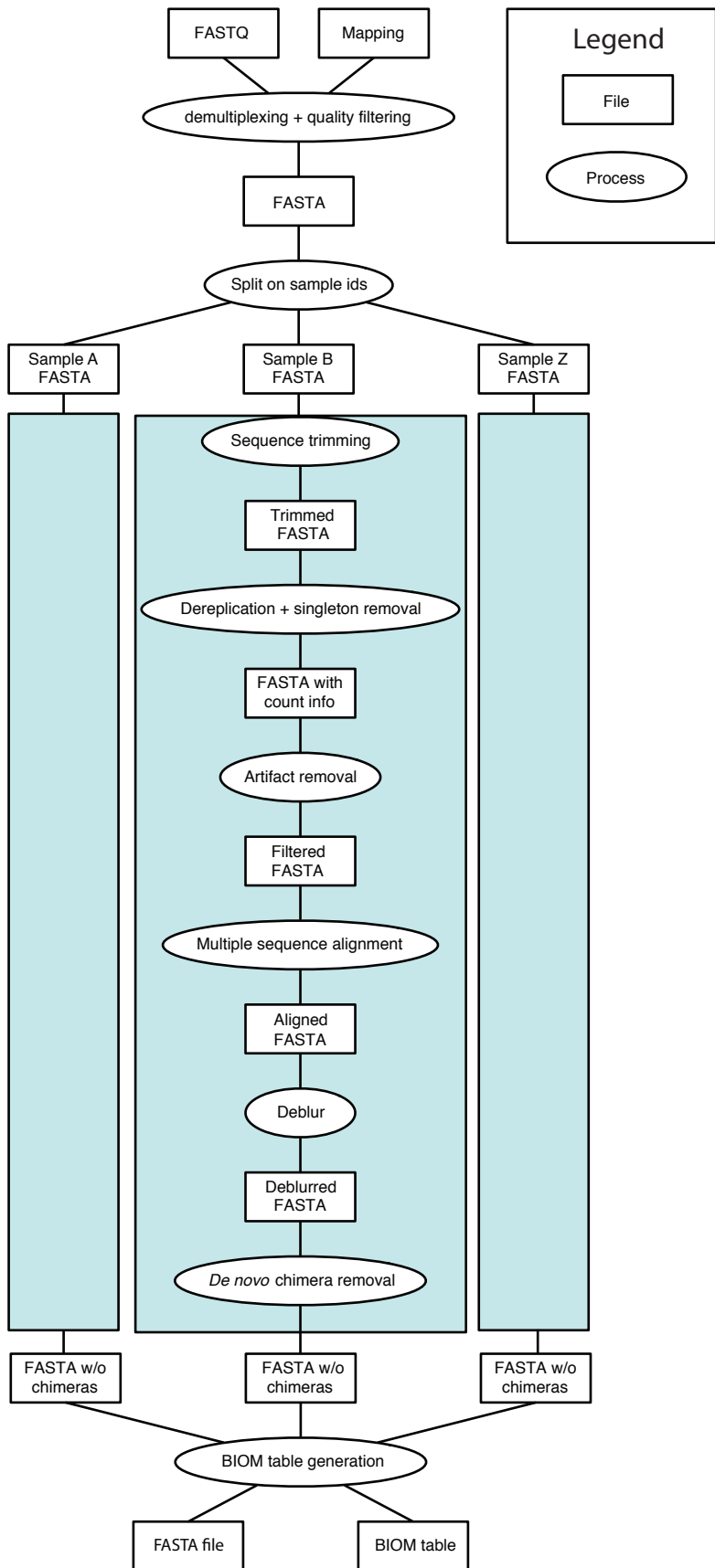

Supplement: FIG S1 [file sys002172091sf1.pdf]

**A**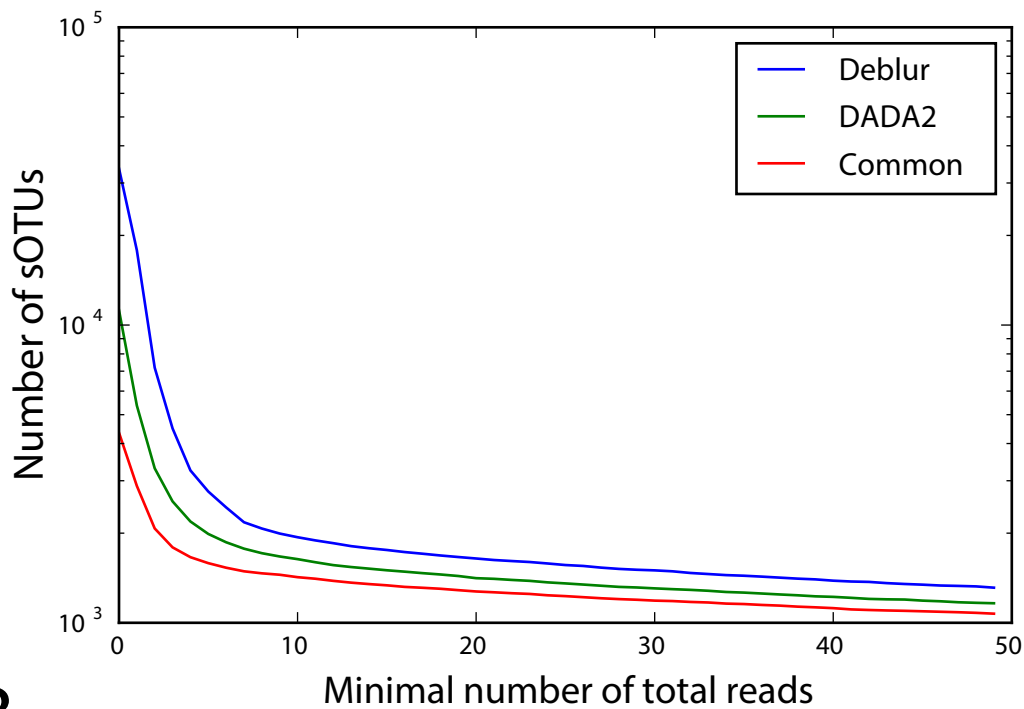**B**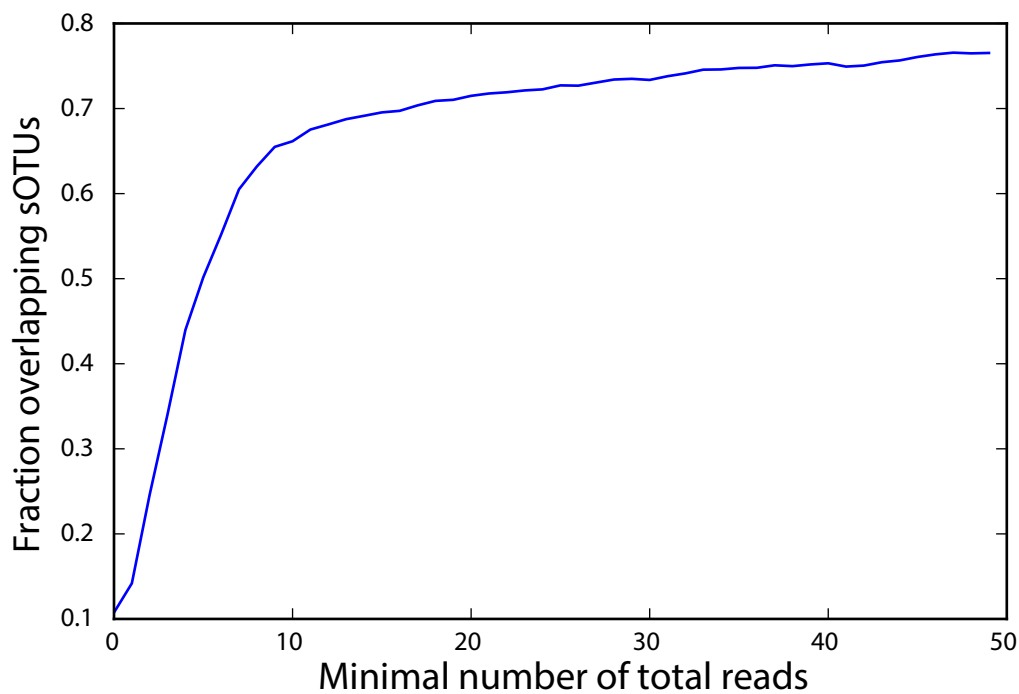

Supplement: FIG S2 [file sys002172091sf2.pdf]

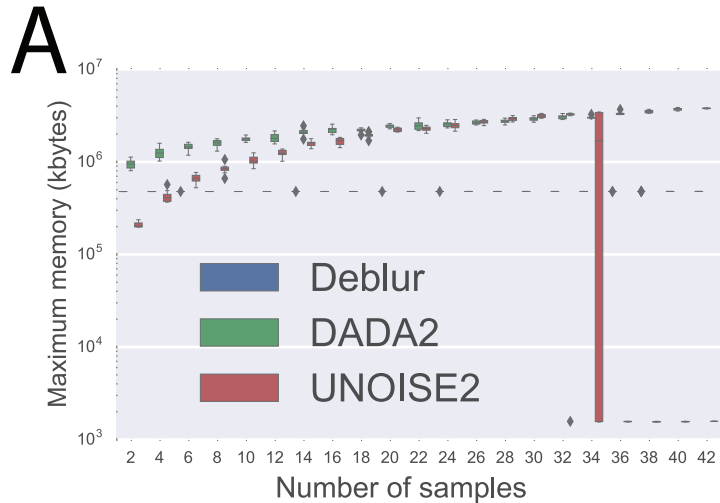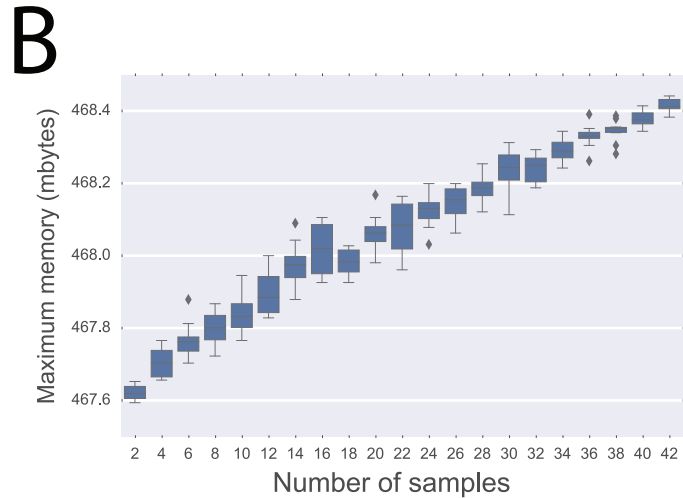

Supplement: FIG S3 [file sys002172091sf3.pdf]
